# Hybrid Dissolving Microneedles Incorporating Hyaluronic Acid Microdepots for Pain-free and Long-acting Corticosteroid Therapy

**DOI:** 10.34133/bmr.0303

**Published:** 2026-01-29

**Authors:** Jae Hwan Lee, Hye-Ju Lee, Geun Jin Song, Dong Kyu Kim, Ahyoung Yoo, Min Lee, Hee Sook Hwang, Chung-Sung Lee

**Affiliations:** ^1^ Department of Medical Science, Soonchunhyang University, Asan 31538, Republic of Korea.; ^2^ Department of Dental Hygiene, College of Health Science, Sun Moon University, Asan 31460, Republic of Korea.; ^3^ Division of Oral and Systemic Health Sciences, School of Dentistry, University of California, Los Angeles, CA 90095, USA.; ^4^ Department of Bioengineering, University of California, Los Angeles, CA 90095, USA.; ^5^ Department of Pharmaceutical Engineering, Dankook University, Cheonan 31116, Republic of Korea.; ^6^ Department of Pharmaceutical Engineering, Soonchunhyang University, Asan 31538, Republic of Korea.; ^7^ Institute for Molecular Metabolism Innovation, Soonchunhyang University, Asan 31538, Republic of Korea.

## Abstract

Inflammatory skin diseases are chronic conditions characterized by persistent inflammation and disrupted skin barriers, necessitating sustained drug delivery. This study presents a long-acting hybrid dissolving microneedle (DMN) patch incorporating hyaluronic acid (HA)-based microparticle depots (TA@MDepots) for intradermal delivery of triamcinolone acetonide (TA). HA is modified to HA acetate to enhance compatibility with hydrophobic TA, enabling efficient microdepot formulation. These TA@MDepots are integrated into the DMN tip layer using mold-casting, resulting in patches with strong mechanical properties and rapid tip dissolution (<3 min) upon application. In vitro and ex vivo studies demonstrate effective skin penetration, sustained dermal retention, and prolonged TA release. The hybrid patch shows excellent cytocompatibility and significantly reduces tumor necrosis factor-alpha and interleukin-6 levels in activated macrophages. In vivo, the patch outperforms commercial TA ointment in a murine ulcer model by accelerating wound closure, reducing epidermal thickening, and lowering inflammatory cytokine expression, despite a lower TA dosage and fewer applications. Histological analyses confirm skin structure restoration and immune modulation, while systemic toxicity assessments show no adverse effects. This hybrid DMN platform offers a minimally invasive, biocompatible, and patient-friendly strategy for prolonged corticosteroid delivery in chronic inflammatory skin diseases and represents a promising alternative to conventional topical or injectable therapies.

## Introduction

Inflammatory skin diseases, including stomatitis, atopic dermatitis, psoriasis, and contact dermatitis, represent prevalent chronic conditions that affect more than 20% of the global population. These disorders are characterized by frequent disease flares, disruption of the epidermal barrier, and sustained cutaneous inflammation, which collectively diminish patients’ quality of life and contribute to increasing socioeconomic burdens [1,2]. Pathophysiologically, immune dysregulation and the excessive release of pro-inflammatory cytokines, including tumor necrosis factor-alpha (TNF-α), interleukin-1β, and interleukin-6 (IL-6), are central drivers of these diseases [3,4]. Standard topical treatments, mainly corticosteroids and calcineurin inhibitors, face challenges such as insufficient skin penetration, frequent application requirements, and potential for local irritation or systemic absorption, particularly with long-term administration [5,6]. Furthermore, traditional topical preparations such as creams and gels are frequently perceived as cosmetically undesirable and may be easily removed by sweating or washing. Consequently, the requirement for repeated application increases the risk of inadvertent overuse and may compromise patient adherence.

To overcome these challenges, microneedle technology has emerged as a promising platform for minimally invasive and efficient intradermal drug delivery [7]. By creating transient microchannels, microneedles can bypass the stratum corneum—the major barrier to transdermal penetration—allowing targeted delivery of therapeutic agents into the viable epidermis and dermis [8,9]. Among various microneedle types, dissolving microneedles (DMNs) fabricated from biodegradable and water-soluble polymers have gained particular attention because of their painless application, complete biodegradability, and ability to encapsulate both small-molecule and macromolecular drugs [7]. These advantages make DMNs especially suitable for the localized and sustained treatment of inflammatory skin diseases, where frequent topical applications often limit patient adherence.

Hyaluronic acid (HA) is a biocompatible and biodegradable polysaccharide widely used in the fabrication of DMNs, particularly for the treatment of inflammatory skin diseases [10]. In recent years, bioactive and biomimetic materials have attracted increasing attention for their roles in regulating cell–matrix interactions and improving therapeutic efficacy, supporting the rationale for employing HA-based polymers in microneedle fabrication [11,12]. Owing to its excellent tissue compatibility, water affinity, and mild immunomodulatory properties, HA provides both structural integrity and biological functionality within dermal environments [13,14]. Unlike synthetic polymers that primarily act as inert scaffolds, HA contributes to skin repair and sustained hydration, making it an ideal matrix material for transdermal drug delivery systems [15,16]. Compared with other water-soluble polymers such as carboxymethylcellulose or gelatin, HA demonstrates enhanced skin permeability, reduced immunogenicity, and superior compatibility with the structural components of the skin extracellular matrix [17,18]. These properties enhance both the microneedle’s ability to penetrate skin efficiently and its mechanical robustness while also promoting the sustained retention of therapeutic agents in the dermal layer when used as a depot-forming matrix. In contrast to previously reported polysaccharide-modified microneedle systems such as pullulan or carboxymethylcellulose, our platform employs partially acetylated HA (HA acetate), which introduces amphiphilic characteristics to the otherwise hydrophilic polymer [19,20]. This structural modification markedly enhances compatibility with hydrophobic corticosteroids, such as triamcinolone acetonide (TA), thereby enabling uniform encapsulation and sustained intradermal delivery [21]. Furthermore, the nonacidic, biocompatible degradation of HA acetate avoids the localized tissue acidification commonly associated with synthetic polymers, underscoring the novelty and clinical relevance of this hybrid platform [22].

Recently, sustained-release delivery systems such as microdepot injections and transdermal patches have been developed to improve patient compliance and achieve long-term therapeutic effects [23]. However, conventional depot injections often cause pain and require medical supervision, which can limit their practicality for chronic skin conditions. In contrast, hybrid microneedle systems provide a less invasive and patient-friendly alternative, offering comparable sustained-release performance while eliminating the discomfort and procedural complexity associated with traditional injections [24].

Microneedles are broadly classified as solid, coated, hollow, dissolving, and hydrogel forming; among these, DMNs are particularly suited to localized, sustained intradermal delivery in inflammatory skin disease [25,26]. Although conventional DMNs offer marked advantages, their rapid dissolution after skin insertion limits sustained therapeutic efficacy [26]. To overcome this limitation, we designed a hybrid DMN system incorporating HA-based microdepots that function as controlled intradermal drug reservoirs while preserving the painless and user-friendly nature of DMNs. Unlike conventional polymeric depots such as poly(lactic-*co*-glycolic acid) (PLGA), which can cause local acidification and tissue irritation, the HA matrix undergoes nonacidic, enzymatic degradation, ensuring excellent biocompatibility for intradermal applications [27]. Furthermore, our platform employs partially acetylated HA acetate, which introduces amphiphilic characteristics that enhance compatibility with hydrophobic corticosteroids and enable sustained intradermal release. This dual functionality—hydrophobic drug compatibility and biocompatible, nonacidic degradation—distinctly distinguishes our HA acetate-based hybrid DMN system from conventional HA or PLGA depots, underscoring its novelty and clinical potential for long-acting corticosteroid therapy.

While various polysaccharide-based microneedle systems have been explored, few have successfully integrated amphiphilic modifications to enable efficient encapsulation and sustained release of hydrophobic corticosteroids [28]. In this context, our platform employs partially acetylated HA acetate, introducing amphiphilic balance that enhances compatibility with TA and provides nonacidic, enzymatic degradation favorable for intradermal environments. This dual functionality—hydrophobic drug compatibility and biocompatible degradation—distinguishes our HA acetate-based hybrid DMN system from conventional HA or PLGA-based depots.

**Fig. 1.**
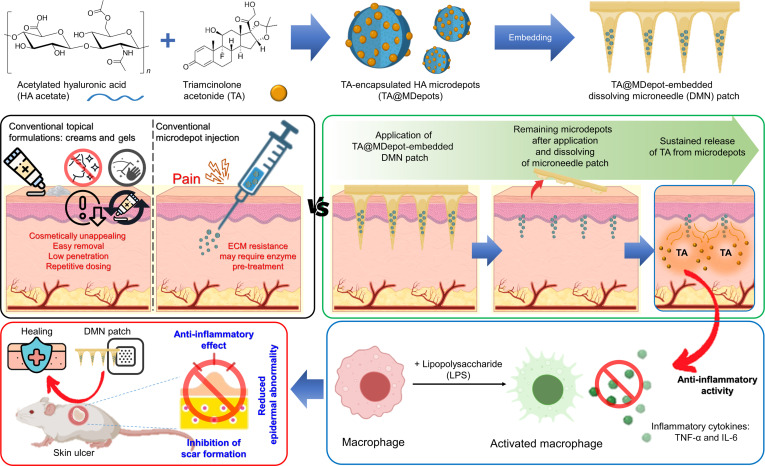
Schematic illustration of a pain-free, long-acting delivery system for triamcinolone acetonide (TA) utilizing a hybrid dissolving microneedle (DMN) patch with hyaluronic acid-based microdepots (TA@MDepots). Unlike traditional microparticle depots that necessitate painful needle-based administration by healthcare professionals, this system allows for patient self-application with minimal discomfort. It also addresses the major shortcomings of routine topical formulations, such as creams and gels, which are limited by insufficient skin penetration, high frequency of administration, and the potential for local irritation or systemic adverse effects. The hybrid microneedle platform achieves efficient intradermal drug delivery and extends therapeutic duration. When applied to inflamed skin, the microneedle tip layer dissolves quickly, thereby releasing TA@MDepots into the subepidermal tissue. These microdepots act as drug reservoirs within the skin, enabling sustained release of TA over an extended period. The prolonged delivery of TA produces significant anti-inflammatory effects, facilitates the healing of skin ulcers, diminishes epidermal irregularities, and suppresses abnormal scar development. This strategy provides a viable alternative to conventional injectable depot therapies, with the potential to enhance patient adherence and substantially decrease procedural pain and anxiety. ECM, extracellular matrix; TNF-α, tumor necrosis factor-alpha; IL-6, interleukin-6.

In this investigation, we introduce a long-acting hybrid DMN patch incorporating HA-based microparticle depots for site-specific and sustained treatment of inflammatory skin diseases (Fig. 1). The hybrid system was prepared utilizing a gentle emulsification–evaporation method and subjected to thorough physicochemical and biological characterization, including mechanical testing, dissolution rate analysis, drug release kinetics, skin penetration assessment, stability during storage, toxicity profiling, and in vivo evaluation of anti-inflammatory efficacy. Our study emphasizes the distinct polymeric benefits of HA in microneedle-mediated delivery systems, demonstrating its promise as a versatile biomaterial for transdermal therapy of persistent inflammatory dermatological disorders.

## Materials and Methods

### Materials

HA (molecular weight 1,300 kDa) was obtained from Purely LY Biotechnology (China). TA was sourced from Tokyo Chemical Industry (Japan). Polyvinyl alcohol (PVA) (86% to 89% hydrolyzed) and pyridine (99+%) were purchased from Alfa Aesar (Massachusetts, USA). Formamide, acetic anhydride, Rhodamine B (RhoB), acetonitrile (high-performance liquid chromatography [HPLC] grade), polyvinylpyrrolidone (K-30), and gelatin were all supplied by Daejung Chemicals (South Korea). Dichloromethane and Tween-20 were obtained from Samchun Chemicals (South Korea). Dulbecco’s modified Eagle’s medium (DMEM), fetal bovine serum (FBS), penicillin–streptomycin, and trypsin were supplied by Welgene (South Korea). A polydimethylsiloxane (PDMS) mold (height: 600 μm; base diameter: 200 μm; array size: 15 × 15) was provided by MPatch (Micropoint Technologies, Singapore). Cell Counting Kit-8 (CCK-8) was sourced from APExBIO (Texas, USA). Lipopolysaccharide (LPS) was purchased from Merck (Darmstadt, Germany). TNF-α and IL-6 enzyme-linked immunosorbent assay (ELISA) kits were provided by Invitrogen (Massachusetts, USA).

### Preparation of acetylation of HA (HA acetate)

To synthesize HA acetate, hydrophobic characteristics were imparted to HA via acetylation with acetic anhydride, following established protocols [20]. Briefly, 2 g of HA was dissolved in 48 ml of formamide with vigorous stirring at 50 °C for 50 min. Next, 12 ml of pyridine and 30 ml of acetic anhydride were added dropwise, and the mixture was allowed to react overnight with stirring at room temperature. Upon reaction completion, the mixture was transferred into an excess of deionized water (DW) and centrifuged at 3,500 rpm for 5 min. This washing procedure was repeated at least 5 times to ensure the efficient removal of unreacted reagents and byproducts. The subsequent precipitate was collected and freeze-dried, yielding HA acetate. The chemical structure of the synthesized product was verified by Fourier transform infrared (FT-IR) spectroscopy and proton nuclear magnetic resonance (^1^H-NMR) spectroscopy in deuterated dimethyl sulfoxide (DMSO-*d*_6_). The synthesized HA acetate was stored in a tightly sealed container at −20 °C and used within 3 months to maintain chemical stability and reproducibility.

### Preparation of TA-loaded HA acetate microdepots (TA@MDepots)

For the preparation of TA@MDepots, a standard oil-in-water (O/W) single emulsion–solvent evaporation technique was adopted, as previously described [7]. In summary, 20 mg of TA and 200 mg of HA acetate were dissolved in dichloromethane to create the organic phase. This solution was added dropwise into 100 ml of an aqueous phase with 2% (w/v) PVA acting as an emulsifier. A 2% PVA solution was used as the emulsifier, and this concentration yielded uniform and stable microdepots, which is consistent with previous optimization results [29]. Emulsification was achieved by homogenizing at 2,000 rpm for 30 min with a high-speed homogenizer (MTops, HM 1200D), generating a stable O/W emulsion. The emulsion was stirred at 400 rpm at room temperature overnight, facilitating complete solvent evaporation. The resulting microdepots were isolated through centrifugation at 3,500 rpm for 5 min (Cryste, Varispin 15), thoroughly washed with DW 5 times, and then freeze-dried. Lyophilized microdepots were stored at −20 °C until analysis.

To quantify TA incorporation in TA@MDepots, 3 mg of microdepots was fully dissolved in acetonitrile. The TA amount was determined using HPLC coupled with ultraviolet detection at 240 nm, referencing a standard calibration curve for TA. The HPLC system comprised a Waters 600 pump (flow rate: 1.0 ml/min), a 600 controller, a 717 autosampler, a Waters 486 ultraviolet detector, and a ZORBAX Eclipse XDB-C18 column (Agilent; 4.6 × 250 mm, 5 μm). The mobile phase utilized was 100% acetonitrile. Encapsulation efficiency was determined from 3 independent experiments (*n* = 3). Encapsulation efficiency was determined by applying the following equation:

$$\begin{array}{c} \begin{array}{c} \text{Encapsulation efficiency}\;\left(\%\right)=(\text{actual drug loading}/\\ \text{theoretical drug loading})\times 100 \end{array} \end{array}$$ (1)

### Fabrication of hybrid DMN patches with TA@MDepots

Hybrid DMN patches containing TA@MDepots were fabricated by a mold-casting approach using a PDMS mold [7]. Initially, the microneedle tip solution was made by dissolving 8% (w/v) PVA and 8% (w/v) sucrose in DW. Approximately 20 mg of TA@MDepots was dispersed in 1 ml of the tip solution with a water-bath-type sonicator (Hwashin Tech, POWER SONIC 510) for 30 min to achieve uniform suspension. Next, 100 μl of the TA@MDepot-loaded solution was dispensed into the PDMS mold and placed under vacuum (around 1 kPa) for 30 s to fill the microneedle cavities thoroughly. The mold was covered and centrifuged at 3,000 rpm for 2 min, facilitating the concentrated migration of microdepots to the tip regions. To ensure consistent loading, the vacuum filling and centrifugation processes were performed 3 times. Excess tip solution was then removed, and a backing layer consisting of 150 μl of 40% (w/v) polyvinylpyrrolidone in DW was applied, allowing it to dry overnight at ambient temperature. Once completely dried, the hybrid microneedles were gently removed from the PDMS mold. As previous reports indicate that ambient humidity compromises microneedle stability, the prepared patches were placed in a desiccator to avoid humidity-driven degradation [30].

### Characterization of TA@MDepots and hybrid DMN patches

Particle size measurement of TA@MDepots was conducted using an optical microscope (Eclipse LV100N POL, Nikon, Japan), with images recorded via an IMT-solution i-Solution camera. For the assessments, TA@MDepots were suspended in DW and distributed uniformly on a glass microscope slide. The diameters of the particles were quantified using the ImageJ software (National Institutes of Health [NIH], USA).

Hybrid DMN patches underwent assessment using a fluorescence microscope (ECLIPSE Ts2-FL, Nikon, Japan). To enable visualization of the microspheres embedded within the microneedle tips, RhoB-loaded microspheres (RhoB@MDepots) were used. Both TA@MDepots and the hybrid DMN patches were further characterized for surface morphology with scanning electron microscopy (SEM; Axia ChemiSEM LoVac, Thermo Fisher Scientific, USA). Samples were mounted on aluminum stubs using carbon tape and imaged directly without conductive coating. For SEM analysis, images were acquired at an accelerating voltage of 15.0 kV.

FT-IR characterization of TA, HA acetate, PVA, sucrose, TA@MDepots, and the hybrid DMN patch was conducted using an FT/IR-4600 spectrometer (JASCO, Japan), with 32 scans accumulated over a spectral range of 500 to 4,000 cm^−1^ at room temperature [7,13]. For each analysis, samples were mixed with potassium bromide (KBr) powder, finely ground, and pressed into pellets. The main purpose of this analysis was to confirm the chemical structure of the synthesized HA acetate. Furthermore, the FT-IR spectra provided insights into possible molecular interactions between TA, HA acetate, PVA, and sucrose within the formulation.

### Microneedle skin insertion study in vitro and ex vivo

To replicate microneedle patch application with microsphere loading, gelatin blocks were fabricated as previously reported [7]. Gelatin powder was dissolved in DW at a concentration of 20% (w/v), poured into a mold, and allowed to solidify. Hybrid DMN patches containing RhoB@MDepots were manually inserted into the gelatin surface by applying thumb pressure for 30 s, after which the backing layer was gently detached. The gelatin matrix was then sectioned to visualize the internal distribution of microspheres using both stereoscopic and fluorescence microscopy.

To assess the insertion efficiency and retention of microspheres in biological tissue, porcine skin sourced from a local slaughterhouse was utilized. Skin samples were stored at −20 °C and subsequently thawed at 4 °C prior to experimentation. Following thawing, the surface was decontaminated with 70% ethanol using lint-free wipes. The skin was stretched and fixed with pins for 30 min to mimic in vivo mechanical conditions. Hybrid DMN patches loaded with RhoB@MDepots were affixed to the stretched skin by applying thumb pressure for 30 s, and after 3 min, the backing layer was gently removed. Microneedle-treated skin was visualized with stereoscopic and fluorescence microscopy (ECLIPSE Ts2-FL, Nikon, Japan) to analyze RhoB@MDepot deposition. For histological evaluation, the treated skin was frozen and cryosectioned into 10-μm-thick sections.

### Stability and dissolution rate of hybrid DMN patches

To assess microneedle stability, hybrid DMN patches containing TA@MDepots were stored in a vacuum chamber at room temperature and at 4 °C. Over a 4-week observation period, changes in morphology and weight of the microneedle patches were quantitatively monitored.

To evaluate the dissolution rate of hybrid DMN patches, porcine skin obtained from a local slaughterhouse was utilized, as detailed in the “Microneedle skin insertion study in vitro and ex vivo” section. The patches were applied to the skin using thumb pressure for specific durations. At designated intervals, the patches were detached from the skin and observed under a microscope. The heights of the remaining microneedle tips were quantified with the ImageJ software (NIH, USA), and the dissolution rate was determined as the proportion of remaining tip height compared to the initial height.

### Mechanical strength of hybrid DMN patches

The mechanical strength of hybrid DMN patches incorporating TA@MDepots was analyzed using a displacement–force test station (EZ-SX, Shimadzu, Japan), following established methodology [7]. To measure compressive strength, an individual microneedle was placed on a rigid stainless-steel platform with its tip facing vertically upward. A flat probe was aligned above the tip and advanced downward at a steady speed of 0.5 mm/min. Force–displacement data were collected from the initial contact point, with the probe lowered by 0.48 mm during the analysis.

### In vitro drug release study of TA@MDepots and hybrid DMN patches

For the evaluation of drug release kinetics from TA@MDepots and hybrid DMN patches, 2.5 mg of TA@MDepots and hybrid DMN patches loaded with an equivalent amount of microdepots were placed separately into individual round-bottom tubes. Each tube received 3 ml of release medium (0.01 M phosphate-buffered saline [PBS] with 0.5% Tween-20). The samples were maintained in a horizontal shaking incubator (BS-21, JeioTech, South Korea) and shaken at 90 rpm at 37.0 °C. At scheduled time points, the tubes were centrifuged at 3,500 rpm for 5 min, and the supernatant was collected and replaced with an equal volume of fresh medium. The tubes were subsequently gently mixed, and incubation continued. Supernatants were analyzed for TA concentration by HPLC as previously described.

### Anti-inflammatory effect of TA@MDepots and hybrid DMN patches

To investigate the anti-inflammatory properties of TA@MDepots and hybrid DMN patches, ELISA analysis was conducted. RAW 264.7 cells were plated in 48-well plates at a density of 1 × 10^4^ cells per well, using 200 μl of complete DMEM containing 10% FBS and 1% penicillin–streptomycin. The cells were incubated at 37.0 °C and 5% CO_2_ in a humidified atmosphere for 24 h. Following this initial incubation, the culture medium was exchanged for complete DMEM supplemented with 0.1 μg/ml LPS and respective test samples. Free TA, TA@MDepots, and hybrid DMN patches were each administered at equivalent TA concentrations. Cells exposed to LPS alone served as the positive control group. After a further 24-h incubation, supernatants were harvested for quantification of TNF-α and IL-6 using ELISA kits according to the manufacturer’s instructions (*n* = 3).

### Cytotoxicity measurement

To assess the cytotoxic potential of microneedle components, the CCK-8 assay was utilized. Both mouse fibroblast (NIH 3T3) and mouse macrophage (RAW 264.7) cell lines were employed in these assays. Cells were maintained in DMEM containing 10% (v/v) FBS and 1% penicillin–streptomycin. NIH 3T3 and RAW 264.7 cells were individually seeded into 96-well plates at a density of 6 × 10^3^ cells per well in 100 μl of complete medium and incubated for 24 h at 37.0 °C in a humidified 5% CO_2_ environment. After incubation, the medium was substituted with a medium containing dissolved test samples, and incubation resumed. The primary-casting solutions for the microneedle and microneedle tips loaded with TA@MDepots were each prepared in complete medium and serially diluted to concentrations of 2,000, 1,000, 500, and 250 μg/ml. Each dose was examined in quadruplicate (*n* = 4). After 24 h of treatment, 10 μl of CCK-8 solution was added, followed by an additional 2 h of incubation. The optical density at 490 nm was subsequently determined using a microplate reader (EL800, Biotek, USA).

### Animal experiments

All animal studies were performed in strict accordance with the guidelines and protocols authorized by the Institutional Animal Care and Use Committee of Soonchunhyang University (Approval No. 2024-0043). Six-week-old BALB/c male mice were supplied by Orient Bio (South Korea). The animals were maintained in a semi-specific-pathogen-free facility under regulated temperature (22 ± 2 °C) and humidity and acclimatized for a minimum of 1 week before the initiation of experiments. Mice were anesthetized with isoflurane during all procedures. Following acclimation, animals were randomly allocated into groups (*n* = 5 per group).

### In vivo retention and degradation of microdepots

To assess the in vivo retention and degradation of microdepots after the administration of hybrid DMN patches, RhoB@MDepot-embedded patches were implemented on BALB/c mice. Before the application of microneedles, each mouse’s dorsal skin was shaved with a depilatory cream to ensure consistent exposure. The hybrid DMN patch was applied to the prepared dorsal skin and pressed gently with a thumb for 1 min to achieve full dissolution of the microneedle tips. Upon removal of the patch’s backing, subepidermal retention of RhoB@MDepots was evaluated using a fluorescence imaging system (Fluor i, NEOscience, South Korea) with controlled excitation intensity and exposure duration. The fluorescence intensity corresponding to the retained RhoB@MDepots was measured quantitatively using the Neoimage software (NEOscience, South Korea).

### In vivo therapeutic study of hybrid DMN patches using a mouse ulcer model

An ulcer model was generated by topical chemical burning, as outlined previously [31]. Anesthesia was induced and maintained with isoflurane (induction: 3% to 4%; maintenance: 1% to 2%) in a controlled mixture of oxygen and nitrogen using an inhalation system prior to any experimental manipulation. Mice were blindly assigned to 1 of 4 groups: an untreated control group, a positive control group (received daily TA ointment application; Oramedy, Dongkook Pharmaceutical Co., Ltd., South Korea), a hybrid DMN patch group with administration every 3 d, and a hybrid DMN patch group treated weekly. Before ulcer induction, the dorsal skin was shaved with a depilatory cream as outlined in the “In vivo retention and degradation of microdepots” section. To induce chemical burns, a 6 × 6 mm paper disk soaked in 90% phenol solution was placed on the shaved dorsal area for 30 s. Each experimental group then received the specified intervention at scheduled intervals. The wound healing progression was periodically monitored, with bright-field images obtained using the fluorescence imaging system (Fluor i, NEOscience, South Korea). On day 14, ulcer severity was assessed based on wrinkle formation, scaling, and hyperpigmentation, with each parameter scored semiquantitatively from 0 to 4 (0, none; 1, slight; 2, moderate; 3, marked; and 4, very marked). The aggregate score (range: 0 to 12) resulted from the sum of the 3 parameter scores [31]. After assessment, mice were euthanized, and skin from the treatment area, along with adjacent tissues, was collected for further analysis.

### Hematology analysis

Blood samples from the experimental groups were obtained via retro-orbital bleeding prior to laboratory analysis. Following collection, samples underwent centrifugation at 10,000 × g for 15 min at 4 °C to isolate serum. Serum biochemical parameters, such as aspartate aminotransferase, alanine aminotransferase, alkaline phosphatase, lactate dehydrogenase, total cholesterol, triglycerides, glucose, lipase, creatinine, and blood urea nitrogen, were analyzed using an automated clinical chemistry analyzer (Hitachi 7180, Hitachi, Japan).

### Histological analysis and immunohistochemical staining

On day 14, dorsal skin tissues from the mice were harvested, fixed with 10% neutral-buffered formalin for 24 h, and embedded in paraffin blocks. Sections 4 μm thick were then prepared and stained with hematoxylin and eosin (H&E) and Masson’s trichrome to evaluate epidermal structure and thickness. Immunohistochemical (IHC) staining was carried out using anti-TNF-α (PA5-120124) and anti-IL-6 (PA5-144595) antibodies (Invitrogen, USA) to assess the inflammatory response. For in vivo biocompatibility evaluation, the heart, liver, spleen, lungs, and kidneys were excised and processed for H&E staining. All slides were analyzed under a stereoscopic fluorescence microscope (ECLIPSE Ts2-FL, Nikon, Japan).

### Statistical analysis

The number of replicates (*n*) for each experiment was as follows: encapsulation efficiency, *n* = 3; in vitro drug release, *n* = 3; dissolution kinetics, *n* = 10; cytotoxicity assay, *n* = 3; cytokine quantification, *n* = 3; and in vivo therapeutic evaluation, *n* = 5. Data are shown as mean ± standard deviation. All statistical analyses utilized the GraphPad Prism 8 software. Statistical significance was evaluated by a 2-tailed *t* test or one-way analysis of variance followed by Tukey’s post hoc test. *P < 0.05, **P < 0.01, and ***P < 0.001 were considered statistically significant.

## Results and Discussion

### Synthesis and characterization of HA acetate

HA, a linear polysaccharide widely utilized in drug delivery systems, is appreciated for its high biocompatibility, biodegradability, and inherent biological activity within skin tissues. However, its restricted solubility in most organic solvents presents a major obstacle for application in single-phase organic reactions, especially those with hydrophobic drugs or polymers. We synthesized a partially acetylated HA derivative, HA acetate, by introducing acetyl groups onto the hydroxyl groups (Fig. S1A). The synthesis was performed in formamide, employing acetic anhydride as the acetylating agent and pyridine as a catalyst. This chemical modification aimed to improve the hydrophobic properties of HA, thus improving its compatibility with hydrophobic substances during microdepot formulation. Related approaches have been used for other polysaccharides, such as HA and pullulan, to enhance amphiphilicity through acetylation [32,33]. It is recognized that the acetylation degree can be modulated by changing the acetic anhydride feed ratio in the reaction mixture [34].

The synthesis of HA acetate was verified via ^1^H-NMR and FT-IR. The ^1^H-NMR spectrum showed a distinct signal for the methyl protons of the acetamido group (–CH_3_) at *δ* ≈ 1.9 ppm (Fig. S1B). The considerable increase in this signal for HA acetate, in comparison to that for unmodified HA, provided evidence of successful acetyl group addition. Calculations based on the peak integration indicated a degree of substitution of about 2.7 acetyl groups per HA disaccharide unit, signifying that both glucose moieties had been modified.

FT-IR spectroscopy serves as an essential technique for elucidating molecular structures and identifying chemical modifications due to its pronounced sensitivity to vibrational changes within functional groups [30]. The magnitude, width, and position of absorption peaks are highly sensitive to shifts in the molecular environment, thereby rendering FT-IR highly effective for verifying structural modifications [35]. Analysis of the FT-IR spectrum of the synthesized HA acetate reveals several distinctive absorption peaks that reflect successful acetylation (Fig. S1C). Notable peaks include a pronounced carbonyl (C=O) stretching band at ~1,751 cm^−1^, a methyl (CH_3_) deformation band near 1,373 cm^−1^, and a signal corresponding to the O–C=O bending vibration at approximately 602 cm^−1^. Of particular interest, the broad absorption feature centered around 3,300 cm^−1^, assigned to the O–H stretching vibration characteristic of native HA, was markedly diminished in HA acetate. This attenuation provides evidence of the efficient substitution of hydroxyl groups by acetyl groups. Collectively, these spectral modifications substantiate that acetylation of hydroxyl groups on the HA backbone was efficiently achieved, producing a more hydrophobic derivative well suited for application in microdepot formulations.

### Fabrication and characterization of TA@MDepots and hybrid DMN patches

**Fig. 2.**
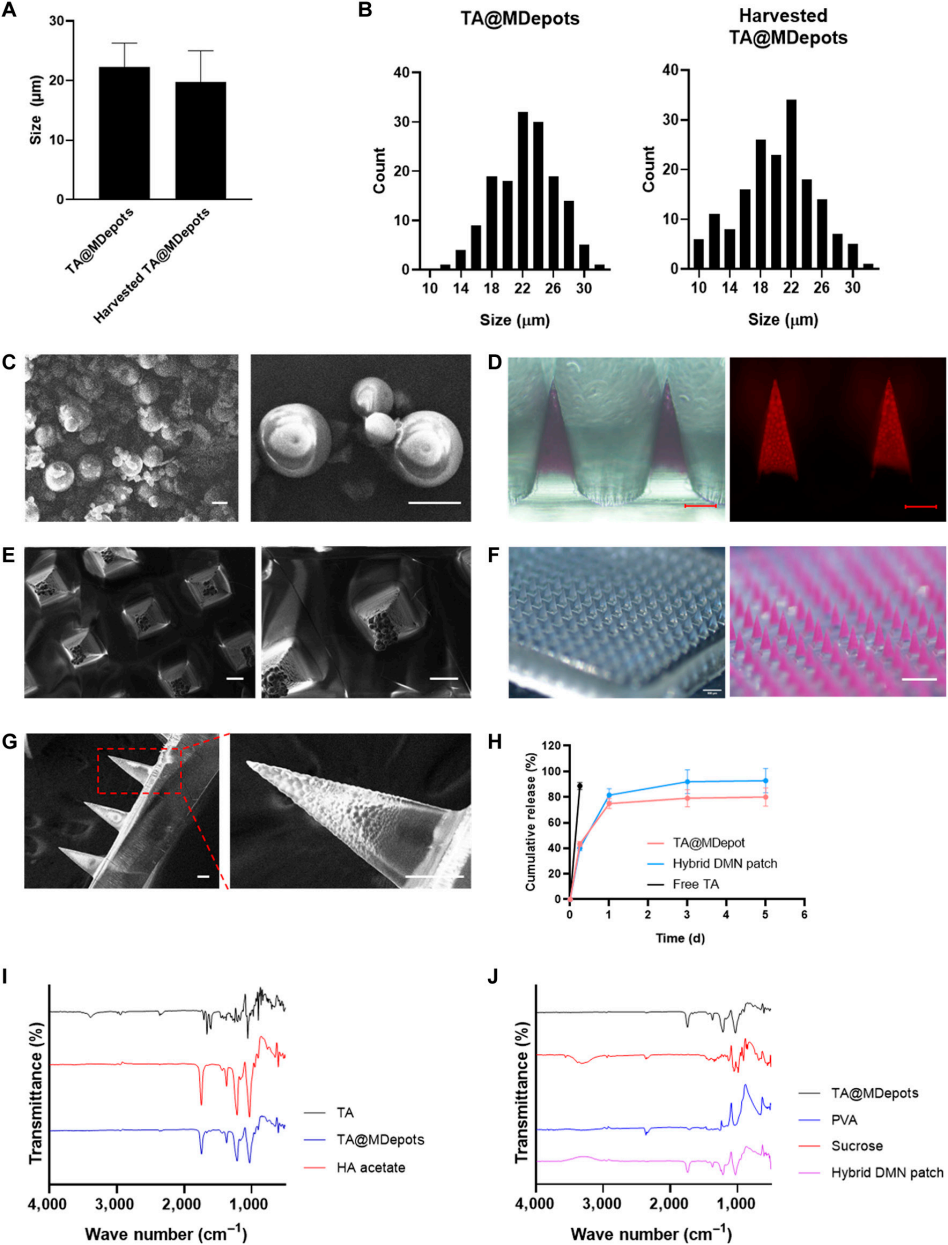
Characterization of TA@MDepots and hybrid DMN patches. (A) Comparison of average particle sizes of TA@MDepots before and after integration into hybrid DMNs. (B) Particle size distributions of TA@MDepots (left) and TA@MDepots extracted from hybrid DMN patches (right) are presented. (C) Shown are representative scanning electron microscopy (SEM) images of TA@MDepots. Scale bars: 10 μm. (D) Optical images depict hybrid DMN patches containing TA@MDepots (left) and RhoB@MDepots (right). Scale bars: 500 μm. (E) Representative SEM images display the top views of cross-sectioned microneedle tips loaded with microspheres. Scale bars: 100 μm. (F) Bright-field (left) and fluorescence (right) microscopic images show hybrid DMN patches containing RhoB@MDepots. Scale bar: 500 μm. (G) Representative SEM images illustrate the side view of a hybrid DMN patch loaded with TA@MDepots. Scale bars: 100 μm. (H) In vitro cumulative release profiles of TA from TA@MDepots, hybrid DMN patches, and the free-TA group (*n* = 3) are shown. (I) Fourier transform infrared (FT-IR) spectra are presented for TA, acetylated hyaluronic acid (HA acetate), and TA@MDepots. (J) FT-IR spectra are also provided for TA@MDepots, polyvinyl alcohol (PVA), sucrose, and hybrid DMN patches. RhoB, Rhodamine B.

Microdepots were produced utilizing a standard O/W single emulsion–solvent evaporation approach, as previously described [7]. Furthermore, to construct hybrid DMN patches incorporating TA@MDepots, a mold-casting technique was applied using a PDMS mold [7]. Particle size measurements indicated that the mean diameter of TA@MDepots was 22.29 ± 4.0 μm, whereas TA@MDepots isolated from the tips of hybrid DMN patches exhibited an average diameter of 19.80 ± 5.2 μm (Fig. 2A and B).

While the diameter of TA@MDepots collected from hybrid DMN patches decreased slightly, statistical analysis revealed no significant difference in size compared to that of the original microdepots. As illustrated in Fig. 2C, TA@MDepots exhibited a smooth, nonporous surface, which is attributable to the exclusion of any porogen during preparation [36]. The encapsulation efficiency of TA in TA@MDepots was determined to be 85.7% ± 7.4%. The hybrid DMN tips were densely packed with TA@MDepots, occupying approximately 75% to 80% of the microneedle tip volume. Based on this loading, each hybrid DMN patch contained approximately 0.01 mg of TA, demonstrating efficient incorporation of microdepots within the microneedle matrix.

The integration of RhoB@MDepots into the tips of hybrid DMN patches was validated using bright-field microscopy, fluorescence microscopy, and SEM (Fig. 2D and E). Both the TA@MDepots (Fig. 2F, left) and RhoB@MDepots hybrid DMN patches (Fig. 2F, right) displayed a 15 × 15 microneedle array within an 8 × 8 mm patch. The microspheres’ restriction to the tip region resulted in a pronounced roughness of the tip surface, yet the needles maintained uniform shape and array structure (Fig. 2G).

The in vitro TA release profiles from TA@MDepots and hybrid DMN patches were monitored over a 5-d timeframe (Fig. 2H). A total of 2.5 mg of microdepots and an equivalent mass of hybrid DMN patches (tip portion only) were immersed in PBS (10 mM) containing 0.5% Tween-20. Both formulation types demonstrated a sustained drug release with no initial burst observed. Conversely, the free-TA control (DMN patch containing unencapsulated TA) exhibited fast release within the first 6 h, emphasizing the microdepot systems’ ability to confer controlled release [37]. Release kinetics did not differ significantly between TA@MDepots and hybrid DMN patches, likely due to the comparable particle size distributions of TA@MDepots.

FT-IR analysis was utilized to examine the structural properties and verify the incorporation of TA into the microdepot formulation. Figure 2I displays the FT-IR spectra of free TA, HA acetate, and TA@MDepots. In the spectrum of pure TA, a prominent absorption band was observed at 3,395 cm^−1^, corresponding to the O–H stretching vibrations of hydrogen-bonded hydroxyl groups, and another at 1,707 cm^−1^, which was assigned to the C=O stretching vibrations from aliphatic ester groups. Additional signature peaks included the band at 1,126 cm^−1^, indicative of the asymmetric stretching of C–O–C bonds in ester functionalities, as well as the band at 1,062 cm^−1^, which described C–F stretching vibrations. These results are consistent with previous literature reports [38]. Although TA represents a relatively minor fraction in the TA@MDepot formulation and some level of spectral overlap with HA acetate is expected, the principal vibrational characteristics of HA acetate remained prominent in the combined spectrum. The lack of substantial peak shifts or the appearance of new absorption bands indicates that TA was physically entrapped within the HA acetate matrix, without evidence of covalent linkage or meaningful chemical interaction. This finding is consistent with the interpretation that TA maintains chemical stability and is allocated in the microdepots via noncovalent encapsulation mechanisms, such as hydrophobic or van der Waals forces, rather than participating in chemical binding.

Further FT-IR characterization was conducted to verify the integration of TA@MDepots into the hybrid DMN patch matrix. Figure 2J presents the spectra of TA@MDepots, sucrose, PVA, and the hybrid DMN patches. The FT-IR spectrum of PVA exhibited fundamental vibrational bands corresponding to its chemical composition. A broad absorption band in the range of 3,550 to 3,200 cm^−1^ was associated with O–H stretching vibrations, indicative of extensive inter- and intramolecular hydrogen bonding. The 2,840 to 3,000 cm^−1^ region demonstrated C–H stretching attributed to alkyl groups. Additionally, the peaks found between 1,750 and 1,735 cm^−1^ were linked to the stretching vibrations of both carbonyl (C=O) and ester-like C–O groups, which likely originate from residual acetate groups in the partially hydrolyzed PVA [39]. Sucrose exhibited its typical FT-IR features, most notably robust absorption between 3,100 and 3,500 cm^−1^, representing O–H stretching vibrations, and a band at 2,920 cm^−1^ for C–H stretching [40]. The FT-IR spectrum of the hybrid DMN patches revealed distinct absorption bands at 600, 1,031, 1,219, 1,378, 1,764, 2,820 to 3,000, and 3,100 to 3,600 cm^−1^, which together encompassed the vibrational characteristics of all major polymers: HA acetate, PVA, and sucrose. Importantly, neither substantial peak shifts nor the loss of major bands was evident, implying that no marked chemical reactions or degradation occurred during microneedle fabrication. Taken together, these spectral attributes demonstrate that TA@MDepots were successfully embedded within the DMN patch matrix, maintaining the structural integrity of all excipients and facilitating a physically stable microneedle platform suitable for transdermal delivery.

### Skin insertion and mechanical analysis

**Fig. 3.**
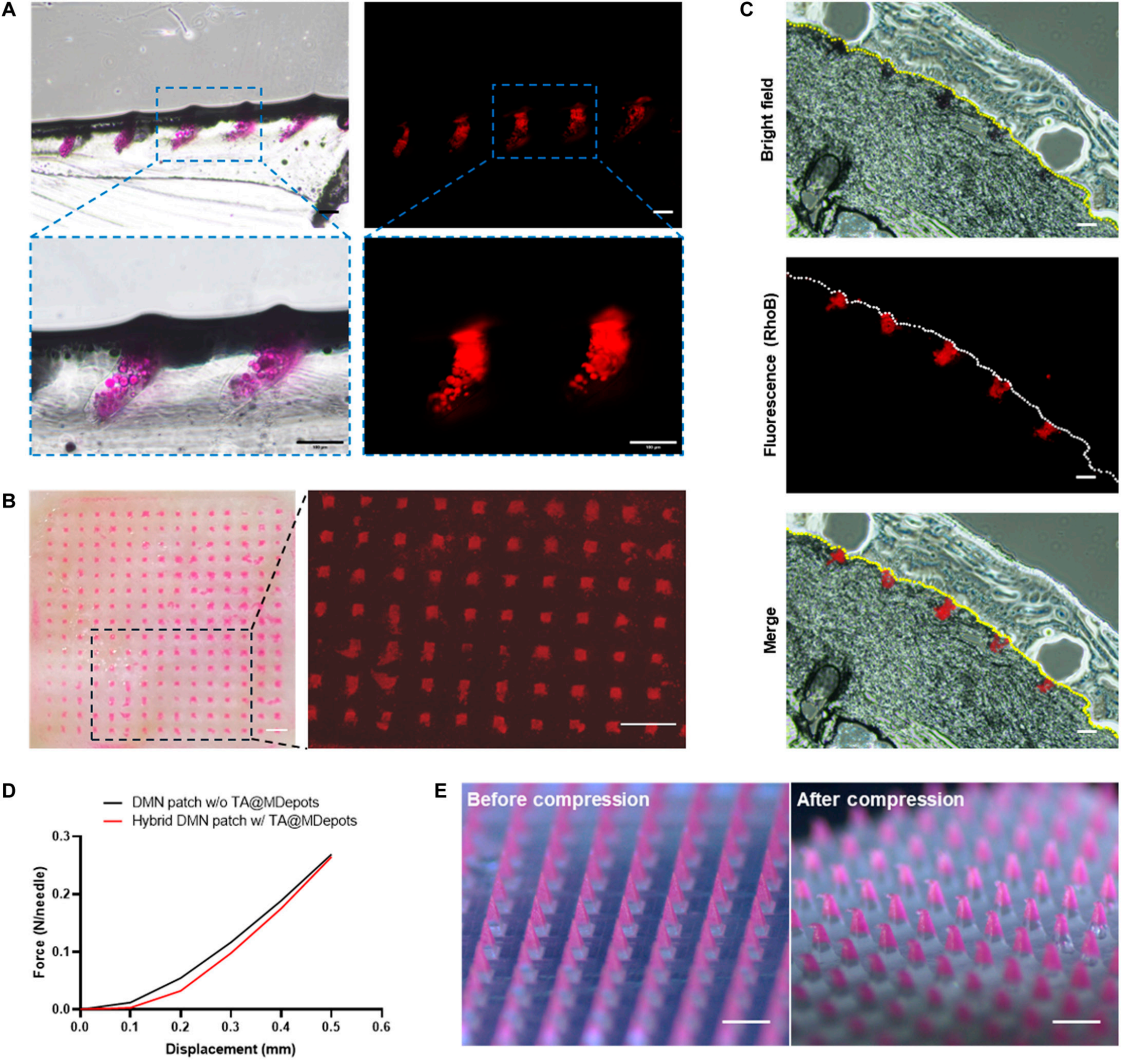
In vitro and ex vivo evaluation of skin insertion and mechanical properties of hybrid DMN patches. (A) Bright-field (left) and fluorescence (right) images of a gelatin block postapplication of hybrid DMN patches encapsulating RhoB@MDepots. Scale bars: 100 μm. (B) Optical (left) and fluorescence (right) images of porcine skin following ex vivo insertion of hybrid DMN patches loaded with RhoB@MDepots. Scale bars: 500 μm. (C) Histological section of porcine skin demonstrating localization of RhoB@MDepots after treatment with hybrid DMN patches. Scale bar: 100 μm. (D) Comparison of the mechanical strength between microdepot-free DMN patches and hybrid DMN patches with TA@MDepots using displacement–force analysis. (E) Representative images showing hybrid DMN patches before and after mechanical assessment. Scale bar: 500 μm.

To simulate the insertion of hybrid DMN patches into skin, a 20% gelatin block was used as a skin analog. RhoB@MDepots were incorporated to enable fluorescence visualization. Patches were applied with thumb pressure for 30 s, after which the backing layer was removed. Imaging of cross-sections confirmed the successful penetration and retention of RhoB@MDepots within the gelatin substrate (Fig. 3A).

For ex vivo assessment, porcine skin was selected because of its close histological similarity to human skin. Applying hybrid DMN patches containing RhoB@MDepots resulted in successful penetration into the subepidermal layer, as demonstrated by both optical and fluorescence microscopy (Fig. 3B). Additional histological analysis demonstrated the persistence of microparticles after removal of the noninserted backing layer (Fig. 3C).

The mechanical properties of hybrid DMN patches incorporating TA@MDepots were evaluated using a displacement–force testing system (Fig. 3D). A decrease in mechanical strength was detected, most likely due to insufficient interfacial adhesion between TA@MDepots and the PVA matrix [41]. Nevertheless, both types of DMN patches endured forces greater than 0.2 N per needle, which substantially exceeds the minimum threshold of 0.058 N documented for successful human skin penetration [42]. Subsequent examination showed only minor tip bending, with no evidence of fracture or severe deformation, demonstrating that the hybrid DMNs preserved adequate mechanical integrity under compressive stress (Fig. 3E).

Collectively, these results demonstrate that the hybrid DMN patch containing microdepots possesses sufficient mechanical robustness to penetrate the skin, notably the stratum corneum, and effectively maintain microdepots within the dermal layer. This capability for intradermal retention may promote extended therapeutic outcomes by enabling prolonged localized drug release.

### Stability and dissolution study of hybrid DMN patches

**Fig. 4.**
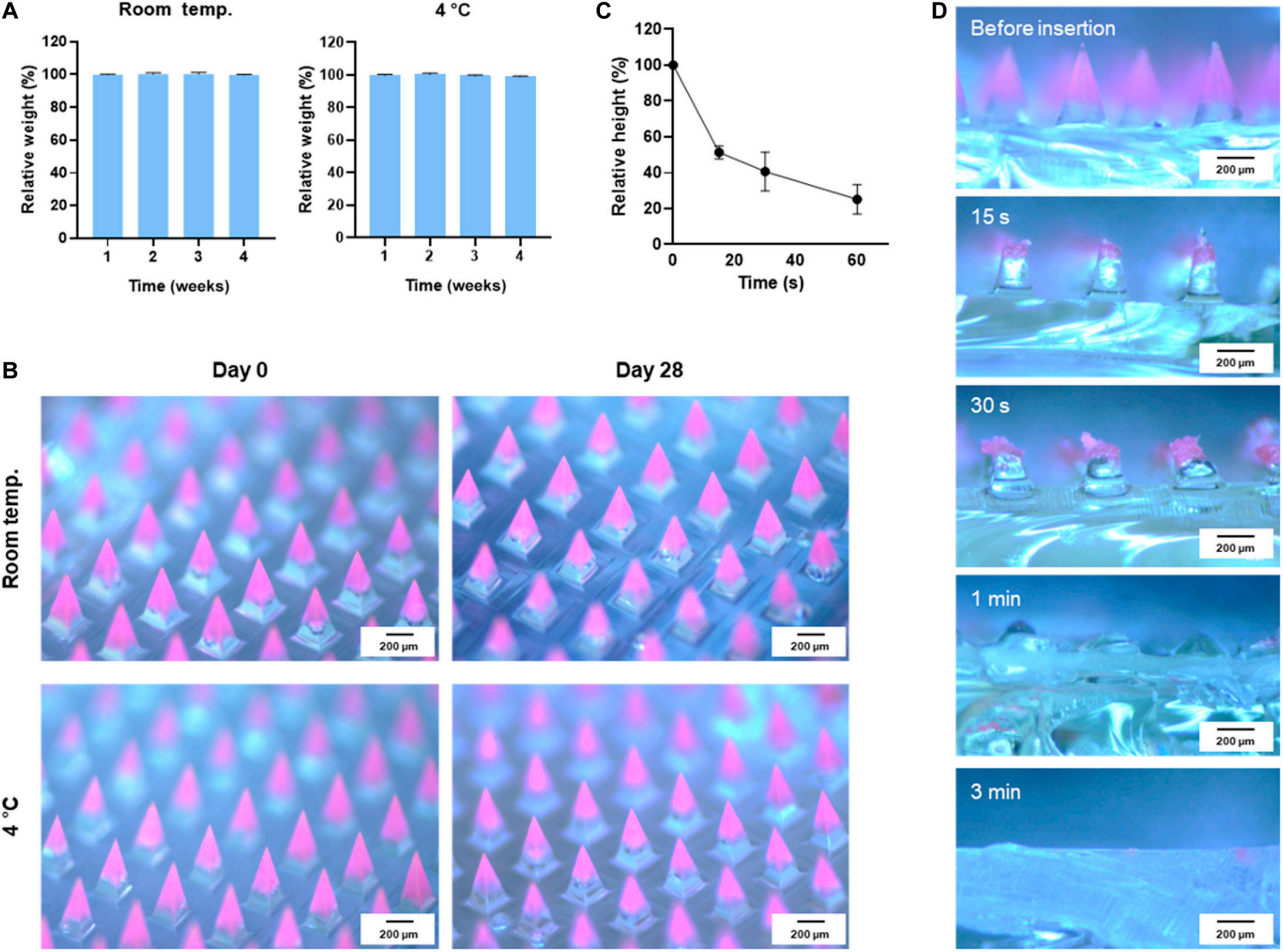
Stability and dissolution characteristics of hybrid DMN patches. (A) The weight variations of hybrid DMN patches were monitored over a 4-week period at both room temperature (left) and 4 °C (right) (*n* = 3). (B) Representative bright-field images of hybrid DMN patches captured prior to and after 4 weeks of storage. Scale bar: 200 μm. (C) Dissolution kinetics of hybrid DMN patches following application to ex vivo porcine skin at specific time intervals (*n* = 10). (D) Bright-field images illustrate the remaining microneedle tips of hybrid DMN patches post-skin application. Scale bar: 200 μm.

To assess the storage stability of hybrid DMN patches containing microdepots under varying temperature conditions, both weight and surface appearance were systematically measured over a 4-week period. For purposes of visual assessment, hybrid DMN patches encapsulating RhoB@MDepots served as the model. Recognizing that humidity can substantially alter DMN dissolution characteristics, all test samples were maintained within a desiccator to minimize variability attributable to moisture [30]. At defined weekly time points, patches were individually weighed and visually examined (Fig. 4A). Throughout the observation period, neither weight nor surface appearance showed meaningful alterations. Additionally, morphological analysis after 4 weeks confirmed the absence of time-dependent degradation or visible structural changes (Fig. 4B). Collectively, these observations indicate that temperatures within the range used for pharmaceutical storage do not substantially compromise the physical stability of hybrid DMN patches. Still, sustained low humidity achieved via vacuum sealing or desiccant use is vital for preserving long-term patch integrity [43].

The dissolution kinetics of the hybrid DMN patches were investigated using ex vivo porcine skin as a model substrate. Patches were applied under thumb pressure at scheduled intervals and then removed for evaluation. The height of the remaining microneedle tips was quantified through ImageJ software analysis (Fig. 4C). After 15 s, about 51.3% ± 3.6% of the initial tip height persisted, indicating an incomplete but substantial dissolution stage. After 1 min, this value dropped to 25.0% ± 8.2%, and the regions loaded with microdepots exhibited pronounced dissolution. By the 3-min mark, no detectable microneedle structure remained, representing complete dissolution (Fig. 4D). The data confirm that hybrid DMN patches rapidly and fully dissolve following skin insertion, thus efficiently delivering the microdepots and associated drug into the cutaneous tissue. This accelerated dissolution supports the feasibility of reducing application duration and potentially enhancing patient adherence and further highlights the suitability of this microneedle-based hybrid system for both immediate and sustained drug release applications.

### In vitro cytocompatibility and anti-inflammatory effects

**Fig. 5.**
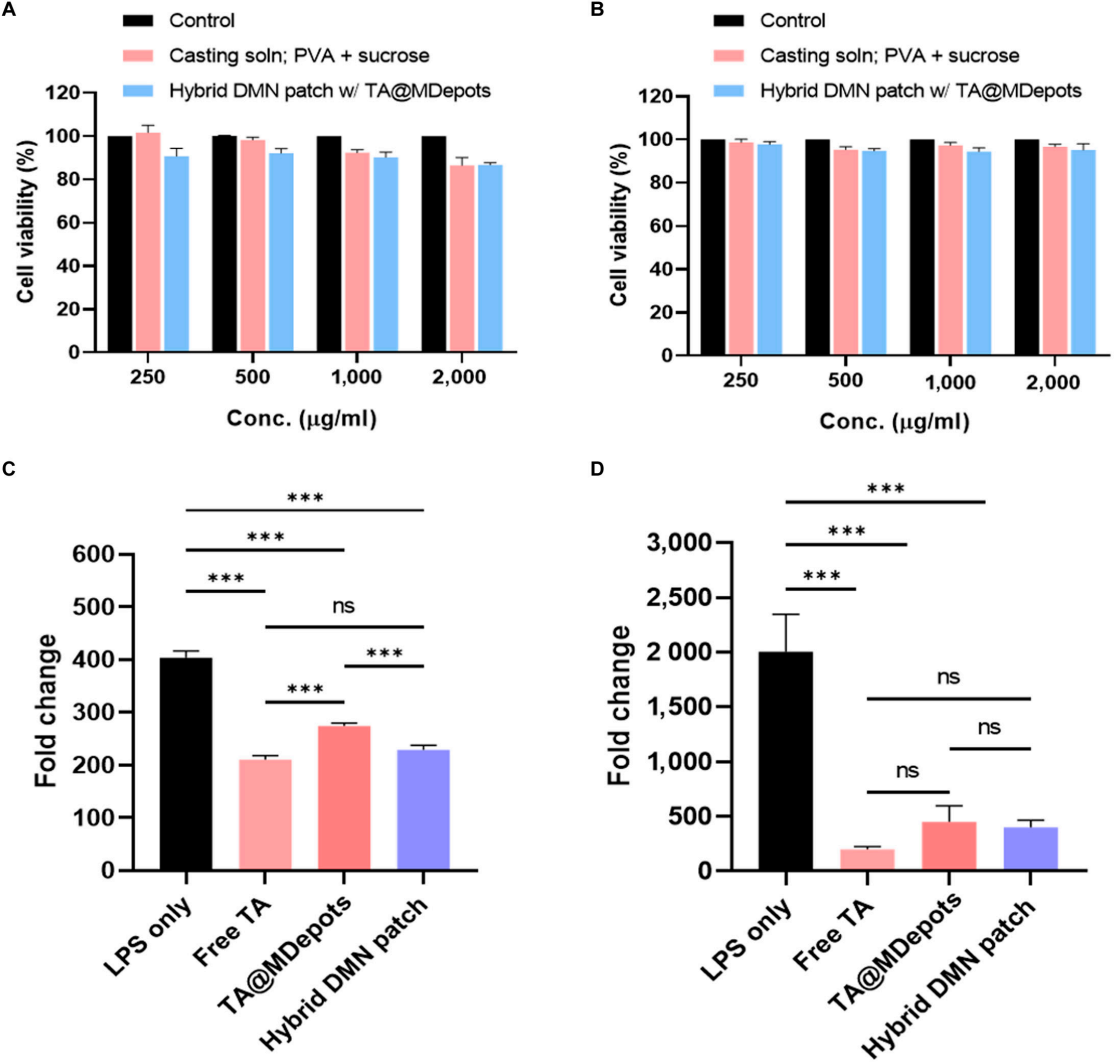
In vitro cytocompatibility and anti-inflammatory effects of hybrid DMN patches. The cytotoxicity of the microneedle casting solution (PVA and sucrose) and microneedle tips containing TA@MDepots was determined using the Cell Counting Kit-8 (CCK-8) assay in (A) NIH 3T3 fibroblasts and (B) RAW 264.7 macrophages, respectively. Data are expressed as mean ± SD (*n* = 3). Quantification of pro-inflammatory cytokines (C) TNF-α and (D) IL-6 from lipopolysaccharide (LPS)-stimulated RAW 264.7 macrophage supernatants following exposure to free TA, TA@MDepots, or hybrid DMN patches. Statistical analysis was conducted using one-way analysis of variance (ANOVA) with Tukey’s post hoc test (*n* = 3; ns, not significant; ***P < 0.001).

The cytotoxicity of hybrid DMN patches, including the components of their microneedle tips composed of TA@MDepots and a PVA-based casting solution, was determined using the CCK-8 assay. As macrophages and fibroblasts are key cellular mediators in both fibrogenesis and inflammatory processes, it was necessary to evaluate the biocompatibility of hybrid DMN patches toward these cell populations [44]. Therefore, NIH 3T3 fibroblasts and RAW 264.7 macrophages were chosen for in vitro assessment. The cells were incubated with different concentrations (up to 2,000 μg/ml) of microneedle tip materials, including both the casting solution (PVA and sucrose) and the TA@MDepot-loaded tips (PVA, sucrose, and TA@MDepots). As indicated in Fig. 5A and B, both NIH 3T3 and RAW 264.7 cells retained high viability across the tested concentration range, with no statistically significant differences compared with untreated controls. These findings establish that the hybrid DMN patch components exhibit no cytotoxicity within the evaluated dose range. This favorable cytocompatibility is primarily attributed to the use of materials such as HA, PVA, and sucrose, which are well documented for their safety and biodegradability [36]. Altogether, these results corroborate the cytocompatibility of the hybrid DMN patch platform and indicate its suitability for safe transdermal administration.

We evaluated the anti-inflammatory properties of hybrid DMN patches loaded with TA@MDepots by conducting ELISAs. RAW 264.7 macrophages were exposed to LPS for 24 h, followed by treatment with either free TA, TA@MDepots, or hybrid DMN patches containing TA@MDepots. After a 24-h incubation period, supernatants were collected from the cultures, and cytokine concentrations were measured. TNF-α and IL-6, the primary pro-inflammatory cytokines produced by activated macrophages, are essential in mediating fibroblast-to-myofibroblast differentiation and perpetuating chronic inflammation [44]. The negative control group received neither LPS nor TA, while the positive control group was exposed to LPS without subsequent therapeutic intervention. As indicated in Fig. 5C and D, TA administration markedly inhibited TNF-α and IL-6 secretion compared to that of the group treated solely with LPS. All TA-treated groups, including those administered free TA, TA@MDepots, or hybrid DMN patches, exhibited measurable anti-inflammatory responses. Although no statistically significant difference was observed between the free-TA and hybrid DMN patch groups, the TA@MDepots and hybrid DMN patch groups displayed slightly less pronounced cytokine suppression than the free-TA group. This observation is likely due to the sustained-release mechanism of TA@MDepot formulations, which provide gradual drug delivery over time, in contrast to the immediate release afforded by free-TA treatment.

In addition to the anti-inflammatory effects of TA@MDepots, we further assessed the intrinsic inflammatory profiles of HA acetate and PLGA-derived acidic byproducts. As shown in Fig. S2, RAW 264.7 macrophages exposed to lactic acid and glycolic acid—a representative mixture of PLGA degradation products—exhibited a pronounced increase in IL-6 secretion, consistent with the localized acidification frequently reported for PLGA-based depots. Indeed, the mixture of lactic acid and glycolic acid reduced the medium pH to below 3, confirming the severe acidification associated with PLGA hydrolysis. In contrast, HA acetate markedly suppressed IL-6 production relative to that in untreated controls, indicating that its enzymatic and nonacidic degradation yields a more biocompatible microenvironment [27]. These results suggest that HA acetate-based depots not only enable sustained corticosteroid delivery but may also inherently minimize inflammation associated with polymer degradation, supporting their advantage over conventional PLGA depots for intradermal applications.

### In vivo retention and biodegradation of microdepots

**Fig. 6.**
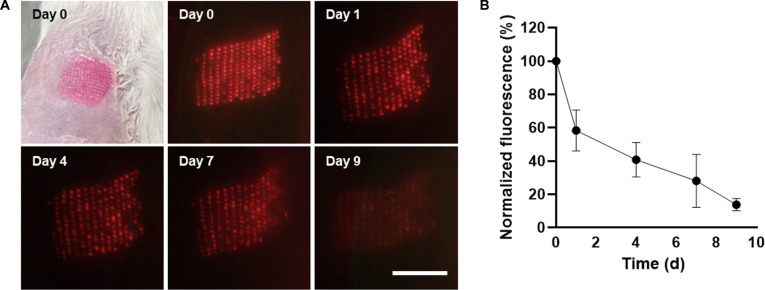
In vivo evaluation of the retention and biodegradation of microdepots administered using hybrid DMN patches. (A) Representative photographs and fluorescence images of mouse dorsal skin after application of hybrid DMN patches containing RhoB@MDepots. Fluorescence imaging was performed at days 0, 1, 4, 7, and 9 following application. Scale bar: 5 mm. (B) Quantitative assessment of fluorescence intensity in the dorsal skin over time, presented as a percentage relative to the intensity at day 0 (*n* = 5).

To assess the intradermal distribution of microdepots following hybrid DMN patch administration, RhoB@MDepot-loaded hybrid DMN patches were applied to the dorsal skin of mice via gentle thumb pressure. After the patch backing was removed, RhoB@MDepots were effectively deposited into the dermal tissue (Fig. 6A). Subsequent in vivo time-lapse fluorescence imaging confirmed the persistence and progressive biodegradation of the embedded microdepots. On day 0, a distinct fluorescent array mirroring the microneedle layout was evident; this signal gradually decreased over the observation period, reflecting continuous dye release and ongoing degradation of the microdepots. Quantitative analysis showed a progressive decrease in RhoB fluorescence intensity, with nearly complete signal disappearance by day 9 (Fig. 6B). Such in vivo sustained retention and degradation aligned with the corresponding in vitro drug release profiles. While the in vitro release profile showed that approximately 80% of TA was released within the first day, the in vivo retention study revealed an only 40% reduction during the same period, indicating that roughly 60% of the drug remained at the application site. The fluorescent signal persisted for over 9 d, confirming gradual depot degradation and sustained intradermal drug availability, consistent with the long-acting therapeutic behavior of the hybrid DMN system. The prolonged persistence of RhoB within the microdepots observed in vivo, compared to in vitro drug release in PBS, is likely due to physiological differences; the in vitro system offers a large-volume sink that enhances diffusion, while the in vivo cutaneous microenvironment has restricted interstitial fluid, resulting in slower diffusion and a more prolonged release profile [36]. Together, these data indicate that intradermal depot persistence under limited interstitial-fluid and non-sink conditions sustains local TA availability for several days, providing a mechanistic basis for the prolonged cytokine suppression observed later in vivo. This difference in release duration between the 2 settings does not compromise the evaluation of long-acting performance but rather reflects the physiological relevance of the in vivo conditions, where restricted interstitial diffusion naturally prolongs depot retention and therapeutic exposure. In addition, these results underscore the long-acting drug delivery potential of our hybrid DMN patch platform and its promise for sustained local intradermal therapy in inflammatory skin diseases.

### In vivo therapeutic efficacy and histological analysis

Given that phenol-induced skin ulcers are associated with acute inflammatory responses, characterized by local immune cell infiltration, increased levels of pro-inflammatory cytokines, and epidermal hyperplasia, this model is widely accepted as a surrogate for evaluating anti-inflammatory strategies in the skin [45]. Therefore, although it is a chemically induced injury model, the ulcer model serves as a pathophysiologically relevant framework for evaluating therapeutic effectiveness in inflammatory skin disorders.

**Fig. 7.**
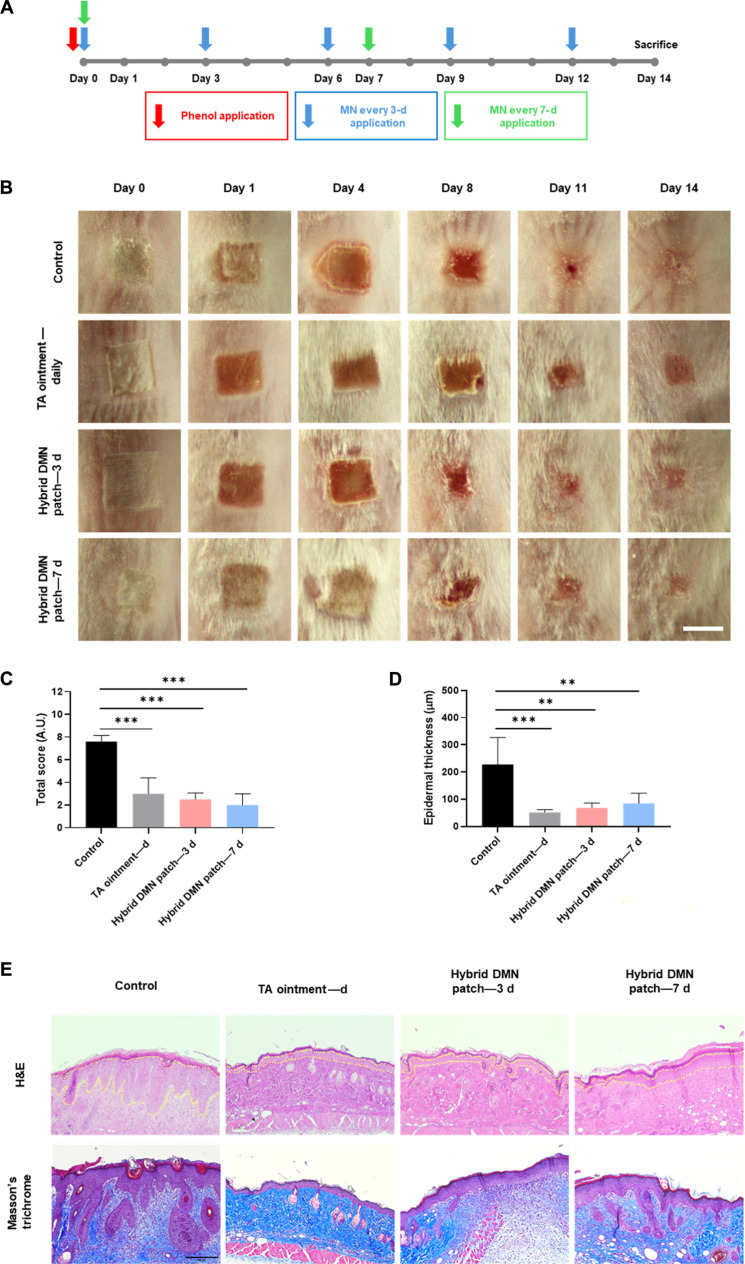
In vivo therapeutic efficacy and histological evaluation of hybrid DMN patches in a murine ulcer model. (A) Schematic timeline depicting the in vivo protocol for the phenol-induced skin ulcer model and subsequent treatment interventions. (B) Representative bright-field images display ulcerated dorsal skin in mice treated by various protocols over a 14-d observation period. Scale bar: 5 mm. (C) Aggregate severity scores determined at day 14, based on assessments of wrinkling, scaling, and hyperpigmentation. (D) Quantitative analysis of epidermal thickness measured from histological skin sections. (E) Representative histological images of hematoxylin and eosin (H&E) and Masson’s trichrome-stained dorsal skin sections. Yellow lines demarcate the interface between the epidermis and the dermis. Scale bar: 200 μm. All values are expressed as mean ± SD (*n* = 5). Statistical analysis was performed using one-way ANOVA followed by Tukey’s post hoc test (**P < 0.01; ***P < 0.001). MN, microneedle.

To evaluate the in vivo therapeutic potential of the hybrid DMN patch system, a mouse skin ulcer model was generated using phenol solution [46]. A 6 × 6 mm filter paper disk saturated with phenol was placed on the dorsal skin of mice for 30 s to establish a localized ulcer, followed by application of the respective treatment groups (Fig. 7A). Commercial TA ointment was applied topically once per day, while hybrid DMN patches were administered at intervals of every 3 or 7 d. As indicated in Fig. 7B, ulcer healing progress was tracked for 14 d. The control group demonstrated marked wrinkling and impaired healing, while groups treated with daily TA or microneedles displayed smoother lesion surfaces and attenuated inflammation. These effects likely result from the anti-inflammatory properties of TA, which inhibit fibroblast recruitment and cytokine production at the wound site [47].

On day 14, severity scores for wrinkling, scaling, and hyperpigmentation were assessed (Fig. 7C), and histological analysis was performed [48]. Both daily-TA-treated and hybrid-DMN-patch-treated groups showed significantly lower severity scores and diminished epidermal thickness compared to controls (Fig. 7D). Notably, the hybrid DMN group received a substantially lower TA dosage (~0.010 mg of TA per patch applied every 3 or 7 d) than the topical ointment group (~0.015 mg of TA per day) yet exhibited comparable wound closure, reduced epidermal thickness, and lower inflammatory cytokine levels. Despite the markedly lower cumulative exposure, therapeutic outcomes were consistent with the retention-enabled delivery described in the “In vivo retention and biodegradation of microdepots” section. Furthermore, the area under the fluorescence–time curve (0 to 9 d) of depot retention negatively correlated with ulcer severity and epidermal thickness at day 14, supporting a clear dose–exposure–response relationship. This comparable efficacy, achieved despite the hybrid DMN group receiving only ~0.01 mg of TA over a 3-d period compared with ~0.045 mg delivered through 3 daily applications of the topical ointment, highlights the superior delivery efficiency of the hybrid DMN system, which ensures precise intradermal targeting and sustained local exposure while minimizing systemic absorption and steroid-related side effects. In addition, its painless and cosmetically acceptable application provides practical advantages over conventional ointments for long-term management of inflammatory skin diseases. These results further highlight the increased intradermal retention and bioavailability of TA from TA@MDepots delivered via hybrid DMN patches, in contrast to the rapid clearance observed with topically applied TA ointments [46].

Despite TA being a relatively inexpensive drug, the hybrid DMN patch offers distinct therapeutic, cosmetic, and practical advantages over conventional ointments. It enables precise, localized intradermal delivery with substantially lower doses, reducing the risk of systemic absorption and steroid-related side effects. In addition, the patch provides a painless and cosmetically acceptable application without the greasiness or visibility associated with topical formulations. Its prolonged intradermal retention minimizes the need for frequent dosing and enhances patient compliance, which is particularly valuable in the long-term management of chronic inflammatory skin diseases.

Histological evaluation provided further validation of these effects. H&E and Masson’s trichrome staining (Fig. 7E) demonstrated that skin from TA-treated mice retained normal architecture, including intact epidermal layers, an organized stratum basale, and visible skin appendages such as sebaceous glands and hair follicles. Conversely, skin from the control group presented marked epidermal hyperplasia, irregular dermal papillae, and loss of skin appendages, all indicative of fibrotic and scarring changes [47]. These histological differences highlight improved tissue regeneration and a reduction in fibrotic remodeling in the TA-treated groups. In addition, histological evaluation at day 14 revealed normal epidermal architecture and the absence of inflammatory infiltration, suggesting that the HA-based microdepots were fully degraded and well tolerated in vivo, with no delayed immune response observed beyond the study period.

Collectively, these results indicate that the hybrid DMN patch acts as a long-acting, intradermal drug delivery platform that effectively accelerates skin ulcer healing, minimizes epidermal pathology, and suppresses excessive scar formation. This multifunctional approach demonstrates therapeutic benefits equivalent to those of traditional topical corticosteroid regimens while substantially lowering overall drug exposure and dosing frequency, factors that have the potential to markedly enhance patient adherence and clinical outcomes for chronic inflammatory skin disorders.

**Fig. 8.**
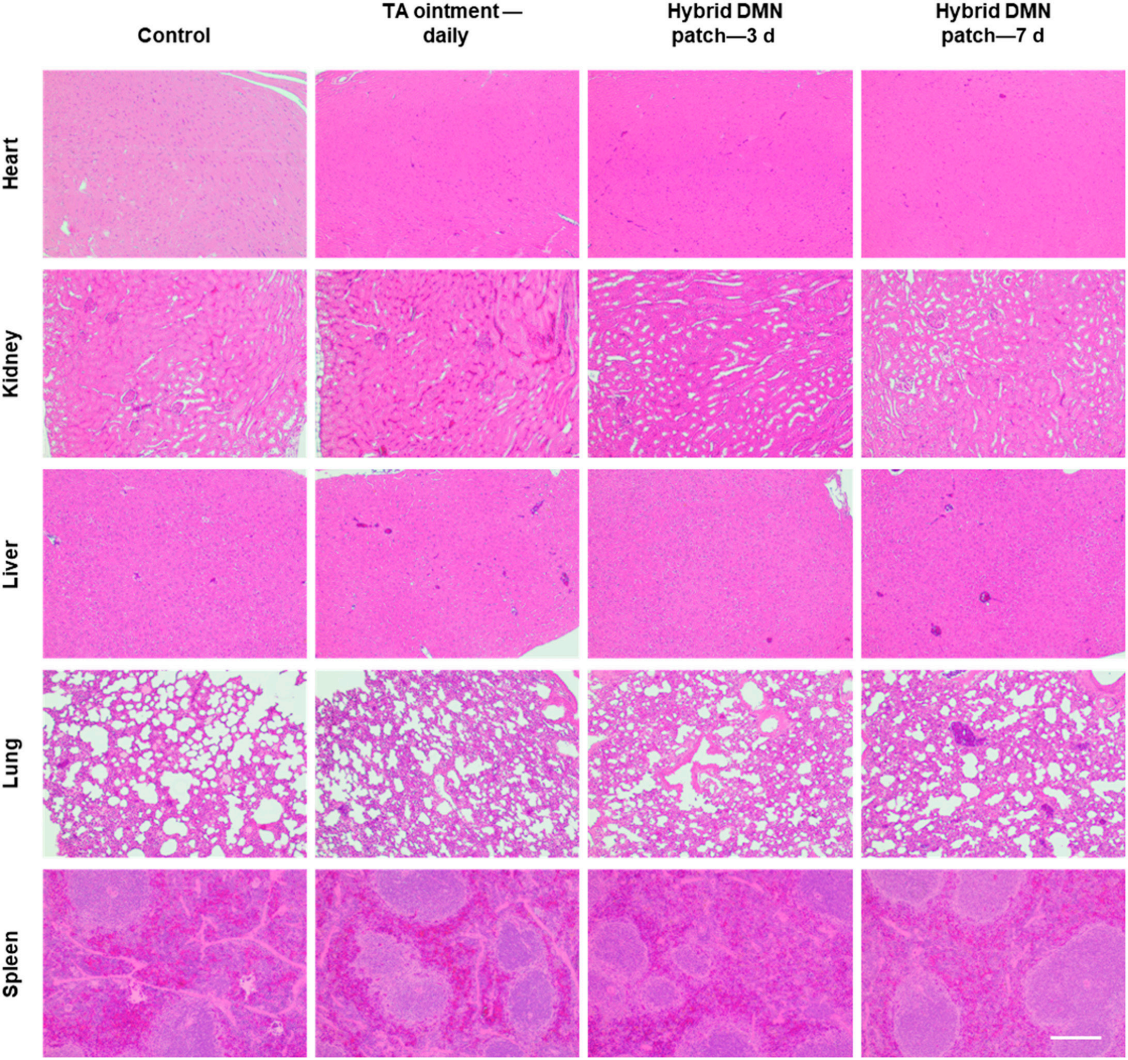
Histological analysis of major organs, the heart, liver, spleen, lung, and kidney, from each treatment group after 14 d of application. Tissues underwent H&E staining. Scale bar: 200 μm.

**Fig. 9.**
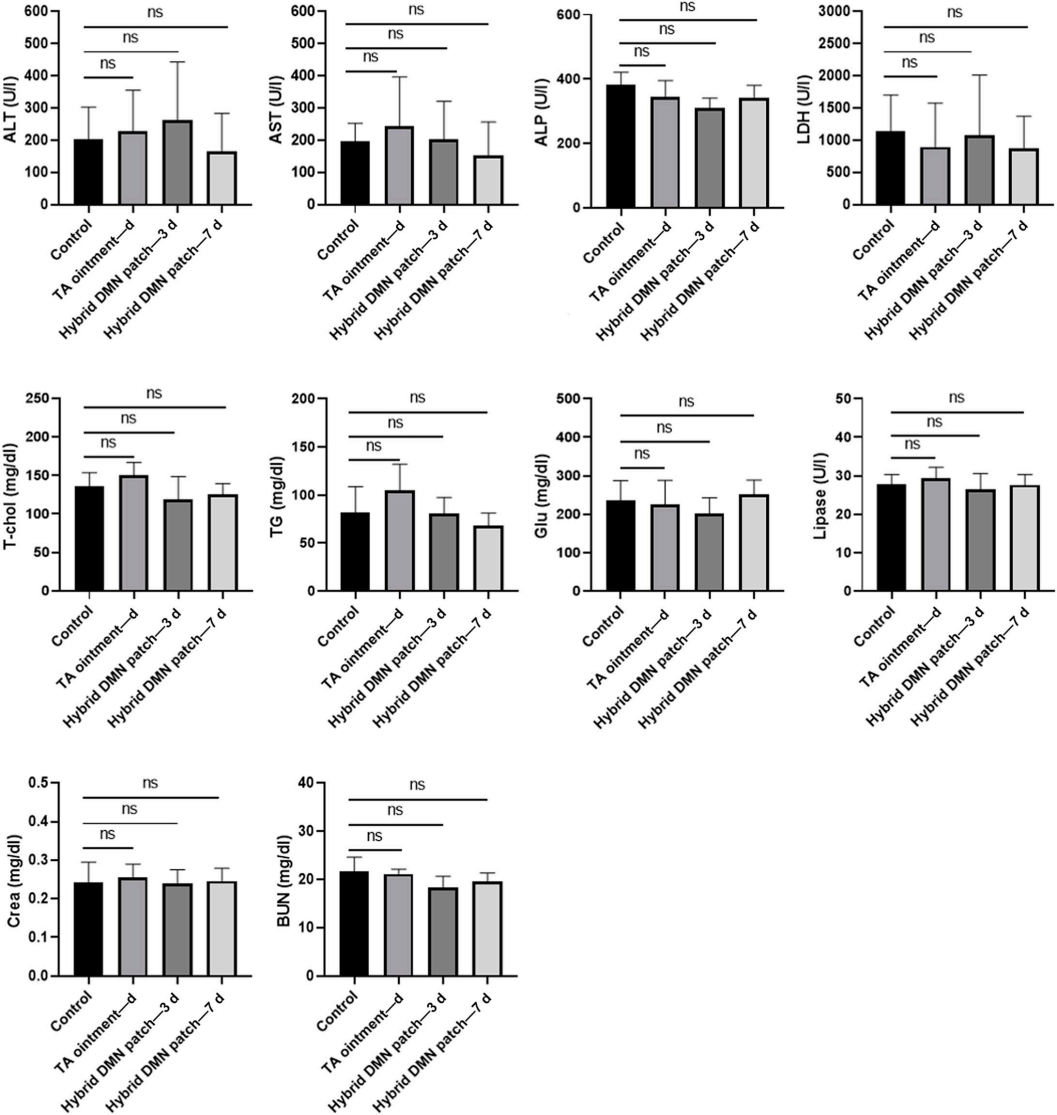
Blood biochemical profiling for liver function markers (ALT, AST, and ALP), lipid metabolism indices (T-chol, TG, and Glu), renal function markers (BUN and Crea), pancreatic marker (lipase), and cardiac marker (LDH) was performed. All measured parameters were within normal physiological limits with no statistically significant differences between treatment and control groups (*n* = 5). Results are expressed as mean ± SD. One-way ANOVA followed by Tukey’s post hoc test was used for statistical evaluation. ns, not significant. ALT, alanine aminotransferase; AST, aspartate aminotransferase; ALP, alkaline phosphatase; T-chol, total cholesterol; TG, triglycerides; Glu, glucose; BUN, blood urea nitrogen; Crea, creatinine; LDH, lactate dehydrogenase.

The biosafety of the hybrid DMN patches was systematically evaluated using both histological and biochemical approaches in microneedle-treated mice. H&E-stained sections of key organs, including the heart, liver, spleen, lungs, and kidneys, demonstrated no evidence of tissue injury, inflammatory response, or structural disruption, confirming the tissue-level biocompatibility of the hybrid DMN patches (Fig. 8). Furthermore, systemic biosafety was further supported by blood biochemical analysis. Key serum markers, including those for liver function (alanine aminotransferase, aspartate aminotransferase, and alkaline phosphatase), lipid metabolism (total cholesterol, triglycerides, and glucose), renal function (blood urea nitrogen and creatinine), pancreatic function (lipase), and cardiac integrity (lactate dehydrogenase), were measured (Fig. 9). There were no statistically significant differences in these parameters between hybrid-DMN-treated and control groups, further confirming the absence of systemic toxicity.

Taken together, these results confirm that the transdermal administration of TA via TA@MDepots incorporated into hybrid DMN patches achieves superior biosafety and systemic compatibility in vivo.

Although microneedles penetrate the stratum corneum, the insertion depth is limited to the superficial dermis, and the needles completely dissolve within minutes after application. This transient penetration causes minimal tissue disruption compared to hypodermic injections. In histological observations, no inflammation, edema, or scarring was detected after microneedle application, confirming excellent skin compatibility even in previously damaged or inflamed areas. In addition to reduced invasiveness, the hybrid DMN system provides practical benefits, including painless self-administration, a low risk of infection, and improved patient compliance for long-term corticosteroid therapy [25,28].

### In vivo anti-inflammatory efficacy of hybrid DMN patches

**Fig. 10.**
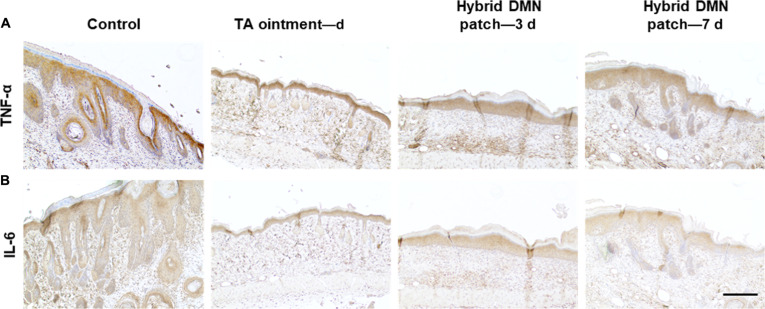
Immunohistochemical (IHC) analysis of pro-inflammatory cytokines in ulcerated skin tissues. Representative IHC-stained sections of mouse dorsal skin were collected on day 14 posttreatment, illustrating the expression of TNF-α (A) and IL-6 (B) among the different treatment groups. Scale bar: 200 μm.

To evaluate local inflammatory responses following treatment, IHC staining for TNF-α and IL-6 was conducted on dorsal skin tissues collected at day 14 posttreatment. As illustrated in Fig. 10, the control group demonstrated pronounced immunoreactivity for both TNF-α and IL-6, signifying persistent inflammation in the ulcerated skin. By comparison, substantially lower cytokine expression was detected in the free-TA, TA@MDepots, and hybrid DMN patch groups. Remarkably, the groups treated with the hybrid DMN patch exhibited similar or even reduced cytokine levels compared to the daily TA ointment group, despite receiving a substantially diminished cumulative TA dose. These results indicate that hybrid DMN patches can efficiently suppress inflammatory cytokine expression via sustained intradermal TA delivery. The IHC data further corroborate the anti-inflammatory efficacy of the hybrid DMN patch system.

The substantial down-regulation of TNF-α and IL-6 achieved with low-dose, infrequent dosing indicates that continuous intradermal release from TA@MDepots effectively modulates the inflammatory skin microenvironment. This localized cytokine down-regulation may play a critical role in not only promoting faster wound closure but also alleviating epidermal pathology and minimizing abnormal scar formation. Overall, these findings underscore the hybrid DMN platform’s therapeutic promise as a prolonged-acting and patient-friendly alternative to standard topical corticosteroid treatments for inflammatory cutaneous conditions.

## Conclusion

In this study, we established a long-acting hybrid DMN patch system containing HA-based microdepots (TA@MDepots) for sustained intradermal TA delivery intended for the treatment of inflammatory skin diseases. TA@MDepots were synthesized successfully, exhibiting high drug encapsulation efficiency, homogeneity, and a nonporous structure. These microdepots were incorporated into microneedle tips by a mold-casting technique, allowing for targeted delivery within dermal tissue. The hybrid DMN patches showed robust mechanical properties, adequate for consistent skin penetration and achieved complete dissolution within 3 min postapplication to ensure effective microdepot deposition. Results from stability testing demonstrated that the patches retained their physical and chemical integrity under standard storage conditions. In vitro release studies and in vivo imaging demonstrated a prolonged drug release lasting several days, supporting the depot effect of the formulation. Anti-inflammatory efficacy was confirmed in LPS-stimulated macrophages, evidenced by decreased TNF-α and IL-6 expression, while both fibroblasts and macrophages exhibited favorable cytocompatibility. In an in vivo mouse ulcer model, the hybrid DMN patches significantly enhanced wound healing, diminished epidermal thickening, and minimized scar formation. Additional histological and IHC assessments demonstrated restoration of skin structure and lower inflammatory cytokine levels in the TA-treated cohorts. Importantly, these therapeutic benefits were realized using a much lower TA dose and reduced application frequency compared to commercially available TA ointment. Extensive biosafety evaluation, including H&E staining of major organs and blood biochemical analyses, found no indications of either local or systemic toxicity. Collectively, these findings position the hybrid DMN patch as a minimally invasive, biocompatible, and user-friendly strategy for sustained corticosteroid administration. This platform has the potential to improve upon conventional topical or injectable therapies by enhancing local efficacy, lowering dosing frequency, and optimizing patient adherence in the treatment of chronic inflammatory skin disorders. In addition, the concept of hybridizing microneedles with commercially available microdepot formulations demonstrates strong translational potential. Such an approach could retain the pharmacokinetic advantages of existing depot formulations while improving patient compliance by eliminating the need for needle-based injection. Therefore, this hybrid microneedle strategy could serve as a clinically practical approach to enhance the usability of current microdepot-type sustained-release formulations.

## Data Availability

The data that support the findings of this study are available from the corresponding authors upon reasonable request.
