# Supplementary material for: Hybrid Dissolving Microneedles Incorporating Hyaluronic Acid Microdepots for Pain-free and Long-acting Corticosteroid Therapy
Source: Biomater Res. 2026 Jan 29;30:0303. doi: 10.34133/bmr.0303 (PMC13268000; doi:10.34133/bmr.0303)
Supplement: Supplementary 1 — Figs. S1 and S2 [file bmr.0303.f1.docx]

*Supporting Information*

Hybrid dissolving microneedles incorporating hyaluronic acid microdepots for pain-free and long-acting corticosteroid therapy

Jae Hwan Lee^a,1^, Hye-Ju Lee^b,1^, Geun Jin Song^a^, Dong Kyu Kim^a^, Ahyoung Yoo^a^, Min Lee^c,d,^*, Hee Sook Hwang^e,^*, and Chung-Sung Lee^a,f,g,^*

^a^ Department of Medical Science, Soonchunhyang University, Asan 31538, Republic of Korea

^b^ Department of Dental Hygiene, College of Health Science, Sun Moon University, Asan 31460, Republic of Korea

^c^ Division of Oral and Systemic Health Sciences, School of Dentistry, University of California, Los Angeles, California 90095, United States

^d^ Department of Bioengineering, University of California, Los Angeles, California 90095, United States

^e^ Department of Pharmaceutical Engineering, Dankook University, Cheonan 31116, Republic of Korea

^f^ Department of Pharmaceutical Engineering, Soonchunhyang University, Asan 31538, Republic of Korea

^g^ Institute for Molecular Metabolism Innovation, Soonchunhyang University, Asan 31538, Republic of Korea

^1^ These authors contributed equally to this work.

* Corresponding authors:

**Chung-Sung Lee, PhD**

Assistant professor

Department of Medical Science

Department of Pharmaceutical Engineering

Institute for Molecular Metabolism Innovation

Soonchunhyang University

Email: chungsung@sch.ac.kr

**Hee Sook Hwang, PhD**

Assistant professor

Department of Pharmaceutical Engineering

Dankook University

Email: hwanghee@dankook.ac.kr

**Min Lee, PhD**

Professor

Division of Oral and Systemic Health Sciences, School of Dentistry

Department of Bioengineering

University of California, Los Angeles

Email: leemin@ucla.edu


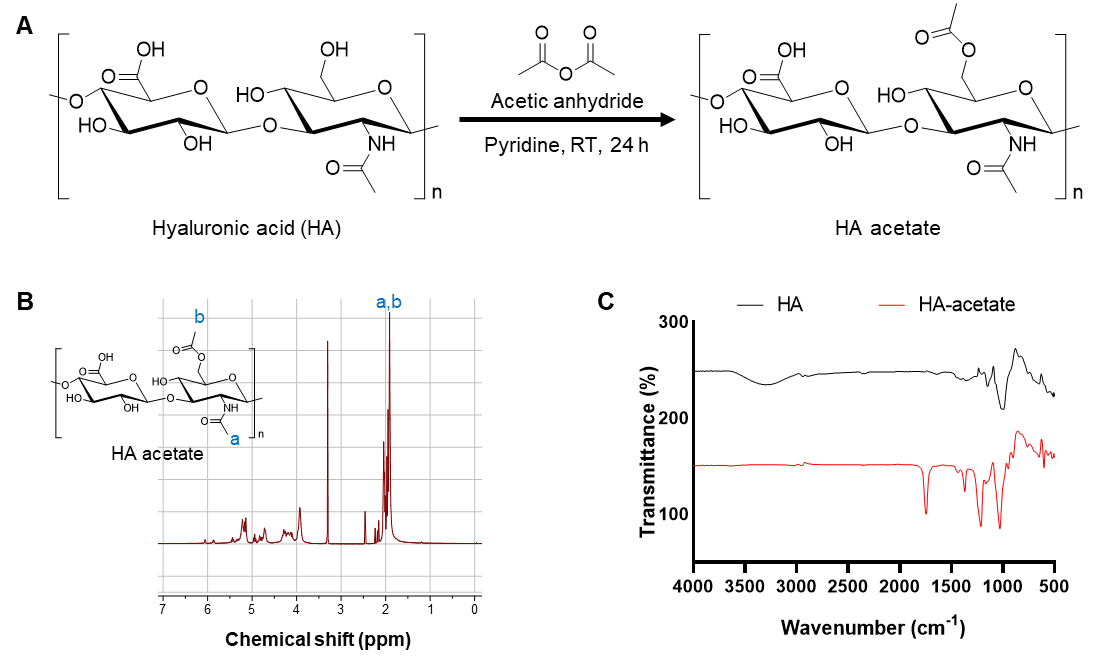


**Figure S1. Synthetic route and structural characterization of acetylated hyaluronic acid (HA acetate).** (**A**) Synthetic route of HA acetate. (**B**) ¹H-NMR spectrum of HA acetate recorded in DMSO-*d₆*. (**C**) FT-IR spectrum of HA acetate.


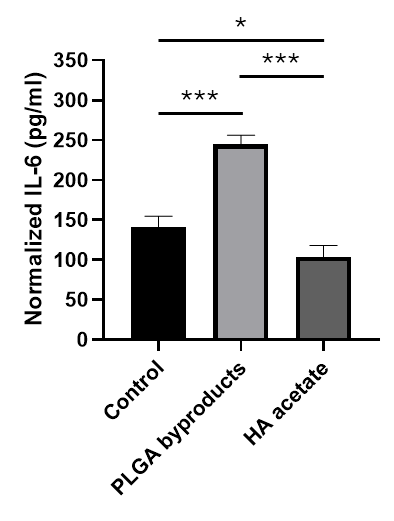


**Figure S2.** Quantification of **IL-6** from RAW 264.7 macrophage supernatants following exposure to PLGA byproducts—lactic acid (0.015 M; 1.35 mg/mL) and glycolic acid (0.015 M; 1.14 mg/mL)—and HA acetate (2.5 mg/mL). The HA acetate sample was prepared by dispersing HA acetate in DMEM and incubating the suspension in a shaking incubator at 37.0 °C and 60 rpm for 7 days. IL-6 concentrations were normalized to cellular metabolic activity measured by the CCK-8 assay to correct for variations in viable cell number. Data are presented as IL-6 levels normalized to CCK-8 metabolic activity (IL-6/CCK-8). The control group represents untreated cells with no added materials. Statistical analysis was conducted using one-way ANOVA with Tukey’s post hoc test (n = 3; **p* < 0.05; ****p* < 0.001).
